# Association Between Cerebrovascular Accident and Vasculitis: Myth or Reality?

**DOI:** 10.7759/cureus.6345

**Published:** 2019-12-11

**Authors:** Nawar Muneer Aljanabi, Sahil S Mamtani, Ashu Acharya, Robins P Gupta Rauniyar, Bilal Haider Malik

**Affiliations:** 1 Internal Medicine, California Institute of Behavioral Neurosciences and Psychology, Fairfield, USA; 2 Medicine, California Institute of Behavioral Neurosciences and Psychology, Fairfield, USA; 3 Surgery, California Institute of Behavioral Neurosciences and Psychology, Fairfield, USA

**Keywords:** vasculitis, stroke, ischemic, steroids, arteries, cerebrovascular accident

## Abstract

This article aims to determine an association between vasculitis and cerebrovascular accidents (CVA) and the ideal management plan to decrease the chances of CVA in vasculitis patients. We also attempt to find a preferred treatment regimen that decreases the complications of CVAs in vasculitis patients, thereby resulting in reduced mortality and morbidity. We reviewed both free-access full-text articles and the abstracts of articles behind a paywall. We used the PubMed database and reviewed 89 articles that matched our inclusion and exclusion criteria. In all, 42 out of the 89 articles had the most relevant data for our article. We used the following keywords to search the database: vasculitis, stroke, cerebrovascular accident, ischemic, arteritis, and steroid. We found an association between subtypes of vasculitis, mostly large vessel vasculitis and CVA. We could not identify a specific cutoff value for specific inflammatory markers that can increase the risk of developing CVA. Besides, there are no formal guidelines for the dosage or the route of administration for corticosteroids, which are the cornerstone of treatment for most vasculitis. We found that male giant cell arteritis (GCA) patients have a higher chance of developing CVA than females, Also interestingly, anemia was found to be protective against CVA development in GCA patients. Sometimes, CVA can happen due to the effects of treatments of some types of vasculitis. We recommend establishing further studies about other subtypes of vasculitis and their associations with stroke.

## Introduction and background

Vasculitis is inflammation caused by the abnormal accumulation of white blood cells in the wall of the blood vessels. The reaction of these cells causes structural damage to the affected blood vessels. Vasculitis can affect any blood vessels. We used the International Chapel Hill Consensus Conference (CHCC) classification that classifies vasculitis into three categories based on the size of the affected blood vessels: large, medium, and small vessel vasculitis [1].

In this study, an attempt has been made to investigate the association between different types of vasculitis and stroke. The association between different types of vasculitis and stroke has been established in many published articles. While one study reported that 3% to 7% of the cases of giant cell arteritis (GCA) did develop stroke, another study conducted at the University Hospital in Turkey showed that 13% of Takayasu arteritis patients developed stroke [2-3]. The incidence of stroke in Takayasu's arteritis (TA) patients ranges between 10% and 20% [4].

Our study also focuses on identifying the most appropriate treatment for stroke in vasculitis patients. Steroids and some immune modulators are a part of the treatment protocol for stroke caused by vasculitis. However, to date, there are no studies or guidelines that can guide clinicians regarding steroid agents that yield good clinical outcomes. Besides, one study showed patients with GCA and anemia had a low risk of stroke development [5]. Based on this finding, we encourage researchers to investigate whether phlebotomy can help in reducing the risk of stroke in GCA patients. Stroke can happen as a complication of vasculitis treatment. Some forms of surgical interventions for vasculitis treatment have been reported to result in stroke as a complication [6].

Identifying a management plan that can reduce the chances of stroke in vasculitis patients will reduce the morbidity and mortality in those patients and also decrease the complexity of their care and the cost of their treatment. We reviewed previously published articles in an attempt to find the best, updated management plans for patients with vasculitis-associated stroke to reduce morbidity and mortality rates.

## Review

We used PubMed database to collect our data. We have not followed the preferred reporting items for systemic reviews and meta-analysis guidelines in our study. We searched the databases using the following keywords: vasculitis, stroke, ischemic, cerebrovascular accident, steroids, and arteritis. We included free full-text human studies in the English language only excluded articles published in other languages. The vast majority of the studies that we searched have been published in the last five years, and most of them were peer-reviewed articles. We followed the ethical and legal protocols while collecting our data. The other limitation was related to vasculitis as a disease. Vasculitis has multiple subcategories, and the studies that are available are not enough to establish the association between each subtype of vasculitis and stroke. Also, some articles required payments to gain full access to their contents, and hence, we reviewed their abstracts alone. 

Stroke

Stroke is still one of the leading causes of death and long-term disability worldwide. Multiple pathophysiological mechanisms lead to stroke as the end outcome, one of them being vasculitis. Vasculitis poses diagnostic and management challenges for healthcare providers. The rarity of some categories of vasculitis in some parts of the world is one of the causes of these diagnostic difficulties. In two studies, we found that TA is more prevalent in Asia and Mexico in comparison with Europe and North America. The annual incidence of TA in North America was estimated to be 2.6:1000000 [7]. The low incidence of TA in North America results in a longer delay in diagnosis and management compared with Asia and Mexico [8-9]. All forms of vasculitis lead to aneurysm formation or stenosis of the affected blood vessels, which leads to impaired blood flow to the vital organs such as the brain.

Giant cell arteritis 

As mentioned in the Introduction section about the classification of vasculitis, the first type is large vessel vasculitis including GCA and TA. In GCA, 3% to 7% of GCA patients developed stroke at one point in their disease course. In most GCA cases, stroke is the cause of death and it has poor prognostic outcomes [10-11]. Most patients develop stroke during the active clinical phase of GCA with a high incidence from the beginning of the development of symptoms until the end of the first month from starting medical treatment with corticosteroids [11]. The posterior circulation of the brain is most likely to be involved, and one study showed that 73% of strokes in the posterior circulation were related to GCA [12]. There are several factors that increased the odds ratio of developing a stroke in GCA patients. Having a history of hypertension, involvement of branches of the ophthalmic artery, or having a high hemoglobin count can significantly increase the odds ratio of getting a stroke. While female gender and a low hemoglobin count will reduce the odds ratio significantly [5]. Corticosteroids still remain the cornerstone in the treatment of GCA and should be started as soon as the patient is suspected with GCA. Early steroid use can significantly reduce the risk of vision loss in these patients. An increase in the risk of developing strokes during the first month of treatment with steroids has been reported. In vasculitis, corticosteroid administration may promote vascular occlusion because corticosteroids do not affect one of the platelet aggregation factors (thromboxane), which leads to an increase in the chances of platelet aggregation and thereby occlusion of the affected blood vessels. Also, the beneficial role of adding antiplatelets is not well established yet [11,13]. But two studies showed beneficial outcomes for antiplatelets aggregation therapy in reducing the risk of stroke in GCA patients [14-15]. Another study attributes the reason for CVAs during the period of steroid initiation to the insufficiency of the steroid dose given to the patients [16]. To date, there are no clear-cut guidelines recommending a specific corticosteroid regimen or route of administration for GCA patients that can reduce the risk of stroke development.

Takayasu's arteritis

It is another subtype of large vessel vasculitis, which mainly affects the aorta and its branches. It appears to be common in East Asia, but multiple cases are reported all over the world. TA has a wide spectrum of signs and symptoms depending on the affected blood vessels and the geographical area of the patient. A study conducted at the University Hospital in Turkey showed different symptoms that TA patients can present with. Symptoms such as claudication and pallor of the extremities, decreased pulsation, and constitutional symptoms were reported by patients who were enrolled in the study [3]. TA has two phases: an active inflammatory phase and a late chronic phase. The early active phase is characterized by general symptoms such as fatigue, fever, night sweats, joint pain, headaches, and skin rashes. During the late chronic phase, vessel involvement is apparent. Those in the late chronic phase may have more localized symptoms including but not limited to renovascular hypertension, acute mesenteric ischemia, retinopathy, amaurosis fugax, and CVA [17].

TA affects the wall of the blood vessels, leading to an increase in the thickness, stenosis, dilation, and/or aneurysm formation within affected vessels [18]. Occlusion of carotid and/or vertebral vessels can lead to neurovascular events, such as stroke and transient ischemic attack [19]. There is no clear pathophysiological mechanism for TA, and there are no specific clinical courses or inflammatory markers that can be specific for TA. Also, there are no clear guidelines to define the disease course or response to treatment. Imaging remains an essential part of disease monitoring. This disease has very severe complications, owing to which early diagnosis and treatment are important for reducing the overall morbidity and mortality of the disease [4].

There are two options for TA treatment: medical and surgical intervention. For medical treatment, the guidelines recommend corticosteroids as the initial treatment for patients with the active phase of the disease. The initial dose should be tapered until withdrawal or to the minimum required dose to control inflammation. Erythrocyte sedimentation rate (ESR) and serum C-reactive protein (CRP) concentrations should be monitored to assess the response to medical treatment. When withdrawal from corticosteroids is difficult, switching to immunomodulators such as cyclophosphamide or azathioprine are possible options. In the case of chronic phase with CRP > 1.0 mg/dL and ESR > 20 mm/h, the administration of steroids should be continued to control systemic inflammation [20]. Approximately half of TA have chronic active phase, and steroids are not enough to provide a remission to the points that healthcare providers can taper the dose of steroids. One study shows that the weekly administration of methotrexate (MTX) with steroids has good clinical outcomes and reduced the adverse effects of steroid use for most patients [21]. Another study showed that adding mycophenolate mofetil results in good clinical outcomes with a very low side effect profile. Mycophenolate mofetil may become a good alternative to steroids and cytotoxic medications. To date, there are no results from any control trials about the use of mycophenolate. Hence, its use should be considered only for patients who do not improve or are not stabilized with conventional therapy [22]. A case report from Japan showed that treatment with infliximab yielded good clinical and laboratory outcomes in patients who were experiencing non-complete control of their disease with steroids and MTX [23]. To date, there are no clear guidelines on preferring certain immunomodulators over the others. Also, there are no studies discussing the role of immunomodulators in reducing the chance of developing stroke in TA patients.

Surgical intervention for TA includes two interventional options: 1) endovascular intervention, including percutaneous transluminal angioplasty and stent-graft placement and 2) open surgical intervention, including surgical bypass grafting, endarterectomy, and graft patch for short segment lesions [24].

Many studies are published on open surgical and endovascular interventions, but there are no studies to show which one of them carries better clinical outcomes. A meta-analysis study showed that patients who underwent open surgical intervention have a higher risk of developing a stroke than patients who underwent endovascular intervention. The location of the lesion plays an important role in determining the risk of stroke development in open surgical intervention. Based on that, the risk of stroke development in the open surgical intervention was higher when the supra-aortic branches were involved rather than the renal arteries [6].

Angiography is the best diagnostic modality to diagnose and determine the disease extent for TA patients. This invasive procedure has a 1.3% to 8.5% risk of developing neurologic or systemic complications [25-27]. There are no clear recommendations reducing the complications associated with angiography. According to a case series from Germany, there is no difference in cerebrovascular involvement between German and Japanese TA patients [28]. There is a huge clinical overlap between GCA and TA. Two studies showed that age, sex, and lesions distribution can be different between the two diseases. Most GCA patients are above 50 years old, while most TA patients are females less than 40 years of age [29]. In addition, almost all parts of the aorta can be invaded in TA. The external carotid artery and its branches are more susceptible to GCA [30].

Primary central nervous system angiitis

Another form of vasculitis associated with CVA is the primary angiitis of the central nervous system. It is an uncommon form of granulomatous vasculitis that affects small-to-medium-sized vessels of the central nervous system specifically. This disease can affect patients between the fourth and the seventh decades of their life with an incident of 2.4:1000,000 [31-32]. Solitary tumor-like mass lesions of primary angiitis of the central nervous system (TLML-PACNS) are one of the subtypes of primary angIitis of the central nervous system. These affect 4% of the patients [33]. The clinical presentation of TLML-PACNS is broad and non-specific. As the disease progresses, multiple neurological deficits happened. Strokes happened in 40% of patients [32]. Many cases were misdiagnosed as a brain tumor or an intracranial infection. Brain biopsy is the gold standard diagnostic modality, and cerebral angiography has low diagnostic power [34]. Magnetic resonance imaging is the preferred imaging in some hospitals, which shows single stenotic lesions in multiple blood vessels [35]. Steroids and immunomodulators are the preferred treatment that yields good outcomes in comparison to steroids alone [36-37]. There are no clear guidelines for the management of PACNS. Also, there are no studies that prefer one immunosuppressive agent over others. One study showed good outcomes of using steroids and cyclophosphamide [34].

Deficiency of adenosine deaminase 2

It is a newly described autosomal recessive syndrome that has a wide spectrum of pathologies such as autoinflammation, vasculitis, and immunosuppression, which leads to broad clinical manifestations such as recurrent fever and multiple cerebrovascular accidents in young patients [38]. It is also associated with portal hypertension, which leads to esophageal varices and puts the patients at risk of hemorrhage.

Deficiency of adenosine deaminase 2 (DAD2) has a very distinct skin manifestation called livedo racemosa, which affects the trunk and extremities. It has a wide branched net-like distribution. Males and females are equally affected. It mostly affects the young age population with 24% of patients who are less than one-year-old age. One study showed that 73% of patients developed this disease during the first decade of their life. Skin and the central nervous system are likely to be affected. Half of the patients have one or more neurological insults sometime in their disease course. Skin manifestations affect 75% of patients. Infections and CVAs are the most common causes of death in these patients. DAD2 has high mortality with 8% of patients died before the age of 30 [39].

Pathophysiology of DAD2

DAD2 causes an alteration in the polarity of macrophages and monocytes and turned them into M1 subtype, which can initiate inflammatory injury and tissue damage. These monocytes affect the integrity of the cell junction of microvascular endothelial cells [40]. We summarized the pathology of DAD2 and the importance of adenosine deaminase 2 in keeping blood vessels intact in Figure 2 below.

**Figure 1:**
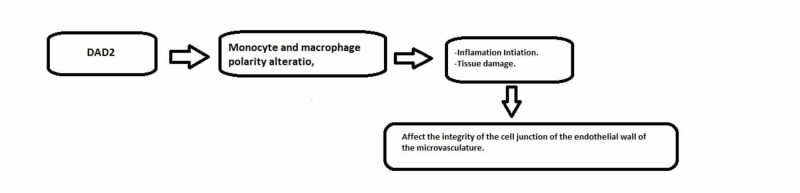
Deficiency of adenosine deaminase 2 pathology that leads to cerebrovascular accidents DAD2, deficiency of adenosine deaminase 2

Management of DAD2

There are no clear diagnostic criteria for DAD2; hence, clinicians should consider DAD2 in their differential diagnoses for patients with non-typical stroke presentations. Serum and plasma testing show adenosine deaminase 2 catalytic activity to be less than 5% in serum or plasma. Besides, molecular testing shows a loss of function mutation in the adenosine deaminase 2 genes [41]. 

For the treatment of DAD2, a study showed that all patients undergoing anti-tumor necrosis factor-alpha (anti-TNFα) therapy did not develop stroke after treatment initiation. However, to date, there are no guidelines that prefer a specific treatment [42].

DAD2 is a newly described disorder; further studies are essential to establish the diagnostic criteria and treatment guidelines. Also, we advise educating healthcare providers about this disease because the treatment plan for stroke due to DAD2 is different from the routine management of stroke due to other causes.

Other forms of vasculitis and their relation to stroke

Other forms of vasculitis are Kawasaki disease and antineutrophil cytoplasmic antibody-associated vasculitis. However, there are not enough articles discussing their association with CVA.

## Conclusions

In this study, we found an association between vasculitis and CVA. But the rarity of some subtypes of vasculitis makes the diagnosis of vasculitis extremely difficult. Also, there are no clinical signs or symptoms that can predict the risk of stroke development in vasculitis patients. Clinicians can predict vasculitis as a cause of stroke in patients who present with an unusual presentation of stroke or develop vertebrobasilar stroke. Surgical intervention as a treatment for some vasculitis subtypes can lead to stroke as one of the treatment complications in these patients. To date, there is no specific management plan that can reduce the chances of stroke in vasculitis patients.
